# Transcriptome expression profiles and regulatory networks in patients with heart failure following myocardial infarction

**DOI:** 10.1186/s12872-026-06424-1

**Published:** 2026-08-12

**Authors:** Yangyang Yuan, Rong Long, Zewen Wei, Lin Ye, Jianying Sun, Shouguo Zhu, Zhi Zhang, Jian Li

**Affiliations:** 1 Department of Cardiology, Anning First People’s Hospital Affiliated to Kunming University of Science and Technology, No.2 Ganghe Rd, Kunming, Yunnan 650300 China; 2 Department of Endocrinology and Metabolic Diseases, Anning First People’s Hospital Affiliated to Kunming University of Science and Technology, No.2 Ganghe Rd, Kunming, Yunnan 650300 China

**Keywords:** Heart failure following myocardial infarction, lncRNA, Translational factors, Whole-transcriptome sequencing

## Abstract

**Background:**

The molecular mechanisms underlying heart failure (HF) following myocardial infarction (MI) (HF-MI) remain incompletely understood, and discoveries from whole-transcriptome profiling hold significant promise for disease prevention and treatment.

**Methods:**

Whole-transcriptome sequencing was performed on whole blood from post-MI patients with and without HF to analyze differentially expressed mRNAs, lncRNAs, and circRNAs, along with functional enrichment analysis. Co-expression networks linking lncRNA-mRNA and circRNA-mRNA pairs, as well as ceRNA networks involving lncRNA-miRNA-mRNA and circRNA-miRNA-mRNA interactions, were constructed for differentially expressed (DE) RNAs. Candidate lncRNAs were validated by RT-qPCR. Transcriptional factor-lncRNA networks were constructed, and candidate relationships were verified using RIP-PCR.

**Results:**

Patients with HF-MI exhibited marked elevation of NT-proBNP and significant reduction in LVEF%. Transcriptome-wide analysis revealed 228 DE-mRNAs, 700 DE-lncRNAs, and 1188 DE-circRNAs in HF-MI compared to non-heart failure patients (NHF-MI). Construction of the ceRNA network identified hsa-miR-6089 and hsa-miR-6771-5p as candidate miRNAs in the lncRNA-miRNA-mRNA network, while hsa-miR-1207-5p and hsa-miR-10392-3p were candidate miRNAs in the circRNA-miRNA-mRNA network. PVT1, SNHG5, TTN-AS1, HCP5, and OIP5-AS1 were identified as the five candidate DE-lncRNAs, with PVT1/SNHG5 showing upregulation and TTN-AS1/HCP5/OIP5-AS1 exhibiting downregulation. The transcription factor-lncRNA network primarily involves GLIS3-HCP5 and ZNF135-PVT1, with validated associative interactions.

**Conclusion:**

This study systematically mapped the regulatory network of HF-MI, suggesting potential involvement of the GLIS3-HCP5 and ZNF135-PVT1 axes in HF-MI, thereby providing a basis for exploring novel therapeutic strategies.

## Introduction

The incidence of cardiovascular disease is more than double that of cancer, and deaths from it are considered the leading cause of mortality in the United States [1]. Among the numerous causes of cardiovascular death, acute myocardial infarction (AMI) accounts for over 50% [1]. Notably, heart failure (HF) is a common complication of AMI, accounting for over 40% of AMI-related deaths between 1998 and 2010 [2]. Subsequent analysis of over 600,000 MI cases showed mortality rates declining to approximately 30%, yet outcomes for AMI patients progressing to HF remain poor [3]. By 2025, studies on one million AMI patients revealed that one in ten individuals required readmission within the first year post-discharge due to HF [4]. Globally, approximately 64 million people are affected by HF, with its prevalence projected to increase by about 46% between 2012 and 2030 [5]. Without guideline-directed medical therapy, the two-year mortality rate for patients with reduced ejection fraction HF is approximately 35% [6]. Multiple agents, including beta-blockers, angiotensin-converting enzyme inhibitors, angiotensin receptor blockers, diuretics, and mineralocorticoid receptor antagonists, are used to treat HF [7]. However, therapeutic outcomes with these drugs are inconsistent, and effects are limited, creating an urgent need for novel molecular targets.

Non-coding RNAs (ncRNAs), including long non-coding RNAs (lncRNAs), circular RNAs (circRNAs), and microRNAs (miRNAs), constitute 98% of the genome. Abnormal expression of ncRNAs in the heart has been found to be associated with various cardiovascular diseases [8]. Research indicates that lncRNA, circRNA, miRNA, and mRNA regulate gene expression through epigenetic, transcriptional, and post-transcriptional mechanisms, as well as by acting as competitive endogenous RNAs (ceRNAs), thereby participating in the development and progression of cardiovascular diseases [9]. Research indicates that lncRNAs undergo significant alterations in HF and play crucial roles in its pathogenesis and reverse remodeling [10]. circRNAs and lncRNAs function as miRNA sponges, regulating multiple mRNA targets and offering novel perspectives for understanding HF pathogenesis and therapeutic strategies [11, 12]. Additionally, ncRNAs enhance inflammation and promote HF progression by modulating transcription factors and epigenetic regulators [13]. These findings suggest that ncRNAs play a crucial role in HF. However, few studies have explicitly elucidated this mechanism.

This study employed whole-transcriptome sequencing to analyze samples from patients with AMI without heart failure (NHF-MI) and those with AMI complicated by heart failure (HF-MI). This study identified differentially expressed (DE) mRNAs, lncRNAs, and circRNAs, constructed interaction networks, screened and validated specific lncRNA candidate biomarkers for HF-MI, and explored their transcriptional regulatory mechanisms. The findings contribute to understanding the mechanisms underlying HF progression after MI and to the development of candidate biomarkers.

## Methods

### Clinical sample collection

This study collected information on non-heart failure patients (*n* = 5) and heart failure patients (*n* = 5) with acute myocardial infarction who visited Anning First People’s Hospital (China) from January 2025 to June 2025. Blood routine tests, thyroid function tests, biochemical indicators, and cardiac echocardiography were performed. Patient inclusion criteria were: (a) AMI diagnosed according to the Universal Fourth Definition of Myocardial Infarction (2018). (b) Symptoms onset within 12 h. (c) Age between 18 and 90 years. (d) Provision of written informed consent. According to the 2024 Chinese Guidelines for the Diagnosis and Treatment of Heart Failure [14], patients must meet at least one of the following criteria [15, 16]: (a) Killip cardiac function classification ≥ Class II; (b) Echocardiographic left ventricular ejection fraction (LVEF) < 40%; (c) Plasma N-terminal pro-B-type natriuretic peptide (NT-proBNP) > 900 pg/mL. The non-heart failure group is defined as Killip Class I with LVEF ≥ 50% and NT-proBNP ≤ 300 pg/mL after acute myocardial infarction. Exclusion criteria: (a) History of chronic heart failure; (b) Concurrent severe hepatic or renal impairment (eGFR < 30 mL/min/1.73 m² or Child-Pugh class C); (c) Concurrent malignancy or life expectancy < 1 year; (d) Concurrent acute infection or inflammatory disease; (e) Participation in another clinical trial within the past 3 months; (f) Incomplete data or inability to follow up. This study was approved by the Medical Ethics Committee of Anning First People’s Hospital (IEC/AF/61 − 03/2023-02.0) and conducted in accordance with the Declaration of Helsinki. Written informed consent was obtained from all patients and/or their families for all samples.

### Whole messenger transcriptome sequencing

Whole-transcriptome sequencing analysis was performed on whole blood samples from patients with non-heart failure (n = 5) and patients with heart failure (n = 5). The sequencing portion was performed by Oebiotech (China). Specifically, RNA extraction was conducted according to the mirVana™ miRNA Isolation Kit (Ambion-1561) protocol. Subsequently, total RNA underwent multiple steps, including ribo-zero depletion and RNA fragmentation, cDNA synthesis, purification, 3’ end adenylation, and adapter ligation using the TruSeq Stranded Total RNA with Ribo-Zero Gold (RS-122-2301) to obtain enriched DNA fragments. One microliter of the sample was loaded onto an Agilent 2100 Bioanalyzer (USA) for library quality control and sequencing. Raw reads underwent data preprocessing: residual rRNA sequences were removed using SortMeRNA [17] software, and redundant reads were filtered via fsatp [18] software to obtain clean reads. Subsequently, Hisat2 [19], RseqQC [20], and StringTie [21] were employed for genomic alignment and transcript assembly of clean reads. The htseq-count [22] software was used to obtain the number of reads mapped to protein-coding genes in each sample and calculate FPKM values. Transcripts longer than 200 bp with ≥ 2 exons were selected as candidate lncRNAs via CPC2 [23], CNCI [24], PLEK [25], and Pfam [26] to identify candidate lncRNAs. circRNAs were identified using CIRI2 [27] software. Differential analysis was performed using DESeq [28] software. Benjamini-Hochberg multiple testing correction was applied, and adjusted p-value (FDR) < 0.05 and fold change > 2 (or < 0.5) as screening criteria. Gene Ontology (GO) and Kyoto Encyclopedia of Genes and Genomes (KEGG) enrichment analyses were performed using Swiss-Prot (http://www.gpmaw.com/html/swiss-prot.html) and KAAS (http://www.genome.jp/tools/kaas/), respectively. miRanda [29] software was used to predict circRNAs and lncRNAs that bind to miRNAs.

### RT-qPCR

RNA was extracted using TRIzol^®^ lysis buffer. The concentration of the 1 µl total RNA sample was measured in an EzDrop1000 spectrophotometer. The first strand of cDNA was synthesized according to the protocol of the riboSCRIPT™ mRNA/lncRNA qRT-PCR Starter Kit (C11030-1, China). PCR cycles were performed on a 7500 Real Time PCR System (USA) using Taq Pro Universal SYBR qPCR Master Mix (Q712-02, China) according to the manufacturer’s instructions. Results were analyzed using the 2^^−ΔΔCt^ method. GAPDH served as the internal control, with primer sequences shown in Table 1.

**Table 1:** Primer sequences

Name	Sequence
GAPDH-F	TTGCCCTCAACGACCACTTT
GAPDH-R	TGGTCCAGGGGTCTTACTCC
PVT1-F	CTTGAGAACTGTCCTTACG
PVT1-R	CAGATGAACCAGGTGAAC
SNHG5-F	GAATGTGACTGACTAGCA
SNHG5-R	GAACTTCCAAGACAATCTG
TTN-AS1-F	ATAGGATATGTGAGAATACC
TTN-AS1-R	GAGGAAGTAGAGTCATTG
HCP5-F	TTACATTACAACTCTGGAA
HCP5-R	CCTTAGATTACCTGTGTT
OIP5-AS1-F	TTATCCTGCTAACATCTC
OIP5-AS1-R	ATTCATACTTGCTACTTCT

### RNA immunoprecipitation (RIP)-PCR

RIP-PCR analysis was performed using the RNA Immunoprecipitation Kit (P0102, Geneseed, China). Specifically, 1 × 10⁷ cells were lysed, and 450 µL of supernatant was incubated with 5 µg of GLIS3 antibody (169792-MSM1-P0, Thermo, USA) conjugated to protein A/G magnetic beads, ZNF135 antibody (bs-7152R, Bioss, China), or Rabbit IgG control (2729, Thermo, USA). Thermo, USA), ZNF135 antibody (bs-7152R, Bioss, China), or Rabbit IgG control (2729, Thermo, USA) at 4 °C for 2 h to overnight. After 5–10 washes with Buffer B/C, elute with Buffer E. Treat the eluate with a DNA removal column and an RNA purification column. Finally, elute the RNA with 30–100 µL RNase-free water for subsequent RT-qPCR detection.

### Statistical analysis

Each group comprised five independent whole blood samples for whole-transcriptome sequencing and clinical analysis, while RT-qPCR and RIP-PCR experiments included at least three independent samples per group. Parametric data are presented as mean ± SD, nonparametric data as median (P25, P75), and dichotomous variables as counts (percentages). Normality was assessed using SPSS (V 27). For comparisons between two groups, parametric data were analyzed using the two-tailed unpaired Student’s t-test, nonparametric data using the Mann–Whitney U test, and dichotomous variables using Fisher’s exact test. The appropriate effect size measure was selected based on the data type (Cohen’s d for parametric data, Cliff’s Delta for nonparametric data, and Cramer’s V for categorical variables).

## Results

### Specificity of clinical indicators in post-MI heart failure patients

This study compared routine clinical venous blood biochemical tests and imaging examination indicators between non-heart failure patients (Con, *n* = 5) and heart failure patients (Case, *n* = 5) following acute myocardial infarction (Table 2). Results showed no significant differences in baseline characteristics such as gender, age, and BMI between the two groups, indicating reliable comparability. There was no difference in comorbidities such as chronic kidney disease between the two groups of patients. Further analysis revealed that NT-proBNP levels in the Case group (6692.00 ± 5327.87) were significantly higher than those in the Con group (254.20 ± 52.99; *P* = 0.027), while LVEF% was significantly lower at 43.80 ± 2.86 compared to 62.60 ± 7.96 in the Con group (*P* = 0.038). Although the adjusted p-value for the NT-proBNP analysis was > 0.05, the large effect size suggests that this finding is exploratory in nature and warrants validation in a larger sample. Furthermore, the study found that diagnostic processes for both groups may not benefit from blood routine and biochemical indicator testing (*P* > 0.05).

**Table 2:** Comparison of clinical data

Type	Indicator		Con (*n* = 5)	Case (*n* = 5)	*P*	*P*.adj	Effect size
Baseline data	Gender	Female	2 (40.0%)	3 (60.0%)	1.000	1.000	0.200
		Male	3 (60.0%)	2 (40.0%)			
	Age		53.80 ± 17.15	52.60 ± 20.91	0.923	1.000	0.063
	BMI		23.56 (21.14, 24.22)	20.57 (20.31, 21.05)	0.421	0.982	0.360
Markers of myocardial injury	MYO		498.00 ± 329.36	585.80 ± 528.12	0.761	1.000	-0.199
	CK-MB (ng/ml)		119.86 ± 102.99	125.10 ± 86.90	0.933	1.000	-0.055
	hs-TnT (ng/ml)		2631.80 ± 2551.30	2641.80 ± 2446.10	0.995	1.000	-0.004
	**NT-ProBNP (pg/ml)**		**254.20 ± 52.99**	**6692.00 ± 5327.87**	**0.027**	**0.473**	**-1.709**
	D-dimer (ug/L)		1.01 ± 0.58	0.39 ± 0.23	0.086	0.604	1.338
Biochemical indicators	ALT (U/L)		25.00 (23.00, 62.00)	31.00 (28.00, 35.00)	0.548	0.992	-0.280
	AST (U/L)		56.00 (49.00, 58.00)	117.00 (92.00, 261.00)	0.421	0.982	-0.360
	Cr (µmol/L)		80.70 ± 14.72	80.10 ± 24.01	0.963	1.000	0.030
	eGFR (ml/min)		82.92 ± 16.53	81.18 ± 31.07	0.915	1.000	0.070
	TC (mmol/L)		4.77 ± 0.78	4.48 ± 1.85	0.753	1.000	0.206
	TG (mmol/L)		2.06 ± 1.07	1.43 ± 0.91	0.342	0.982	0.638
	LDL (mmol/L)		2.96 (2.88, 3.05)	2.02 (2.01, 3.23)	1.000	1.000	0.04
	HDL (mmol/L)		0.98 ± 0.25	1.31 ± 0.36	0.131	0.762	-1.065
	Uric acid (mg/dl)		405.00 ± 114.03	381.80 ± 145.24	0.802	1.000	0.175
	HbA1c (%)		8.20 ± 2.83	6.68 ± 1.31	0.366	0.982	0.691
	CRP (mg/L)		40.10 ± 52.64	4.53 ± 4.34	0.308	0.982	0.952
Complete blood count	WBC (10^9/L)		8.72 (8.52, 11.82)	12.35 (9.9, 12.50)	0.530	0.992	-0.280
	Neutrophil		9.42 ± 4.24	9.67 ± 2.16	0.907	1.000	-0.076
	Lymphocyte		0.83 ± 0.29	1.23 ± 0.54	0.187	0.934	-0.913
	Monocyte		0.96 ± 1.10	0.42 ± 0.25	0.309	0.982	0.687
	Platelet		210.60 ± 58.11	251.80 ± 66.62	0.328	0.982	-0.659
	HB		143.40 ± 16.64	135.00 ± 26.67	0.567	0.992	0.378
Echocardi-ography	**LVEF (%)**		**62.60 ± 7.96**	**43.80 ± 2.86**	**0.001**	**0.038**	**3.144**
	LADd		32.80 ± 3.70	30.00 ± 3.67	0.264	0.982	0.759
	LVDd		44.40 ± 3.21	44.80 ± 1.48	0.807	1.000	-0.160
	IVST		11.06 ± 0.93	9.96 ± 0.64	0.062	0.538	1.374
	LVPWT		10.16 ± 0.44	9.96 ± 0.34	0.449	0.983	0.503

Bold indicates significant differences

*BMI* body mass index, *MYO* myoglobin, *CK-MB* creatine kinase-MB isoenzyme, *hs-TnT* high-sensitivity troponin T, *NT-ProBNP* N-terminal pro-brain natriuretic peptide, *D-dimer* D-dimer, *ALT* alanine aminotransferase, *AST* aspartate aminotransferase, *Cr* creatinine, *eGFR* estimated glomerular filtration rate, *TC* total cholesterol, *TG* triglyceride, *LDL* low-density lipoprotein cholesterol, *HDL* high-density lipoprotein cholesterol, *HbA1c* glycated hemoglobin A1c, *CRP* C-reactive protein, *WBC* white blood cell, *HB* hemoglobin, *LVEF* left ventricular ejection fraction, *LADd* left atrial diameter in diastole, *LVDd* left ventricular end-diastolic diameter, *IVST* interventricular septal thickness, *LVPWT* left ventricular posterior wall thickness

P.adj, Corrected *P*-value (FDR-BH)

### Whole transcriptome expression profiling characteristics of HF-MI

**Fig. 1:**
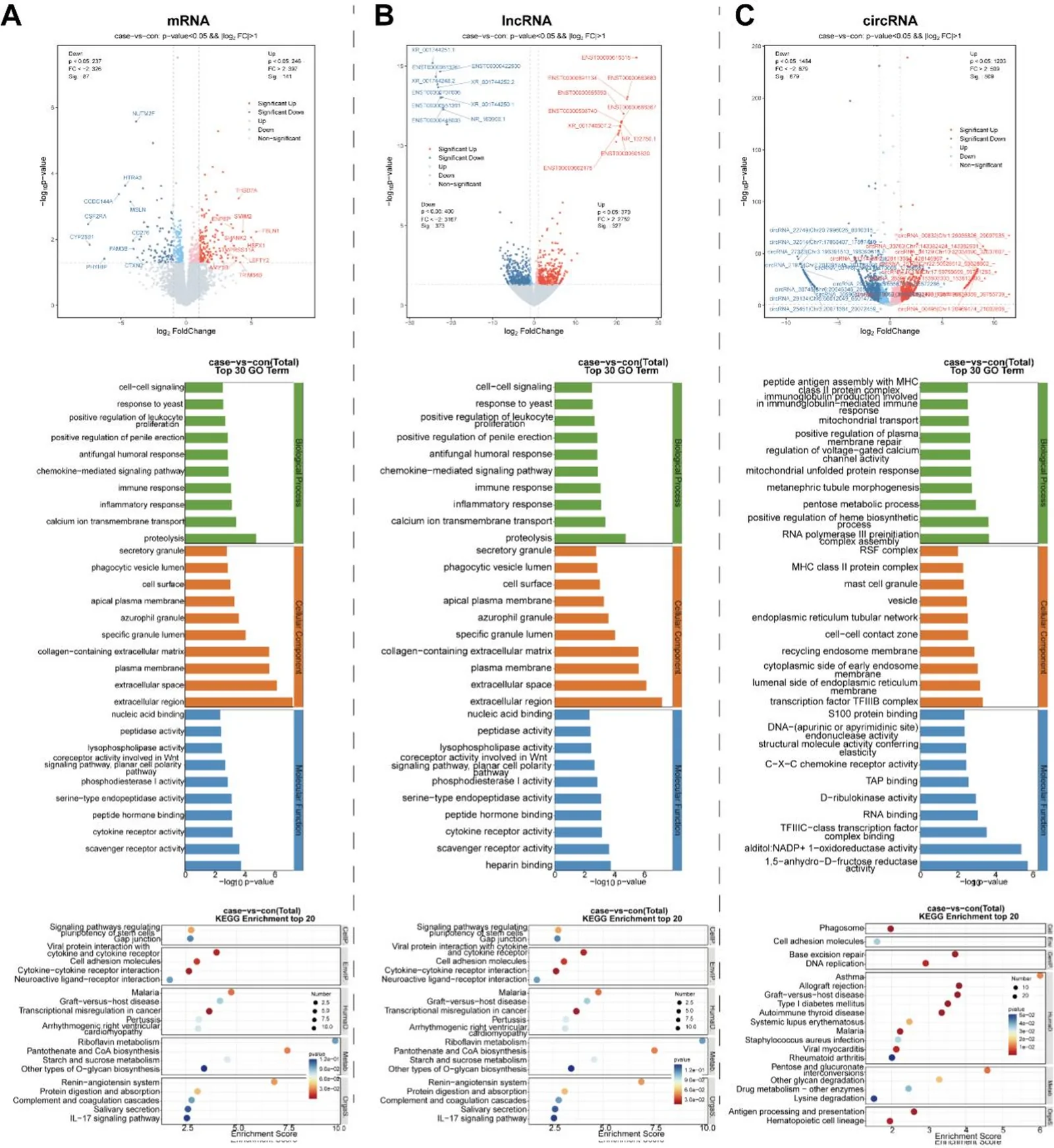
Differential transcriptome and functional enrichment analysis in non-heart failure and heart failure patients after acute myocardial infarction. **A**: Differential mRNA volcano plot and GO/KEGG functional enrichment analysis in non-heart failure and heart failure patients after acute myocardial infarction (top to bottom). **B**: Differential lncRNA volcano plot and GO/KEGG functional enrichment analysis in non-heart failure and heart failure patients after acute myocardial infarction (top to bottom). **C**: Volcano plot of differentially expressed circRNAs and GO/KEGG functional enrichment analysis in non-HF and HF patients after acute MI (top to bottom). *n* = 5. Screening criteria: *p*Value < 0.05 and fold change > 2 (or < 0.5)

This analysis completed whole transcriptome sequencing for 10 samples, yielding a total of 128.42 G of CleanData. The effective data volume per sample ranged from 11.73 to 13.62 G, with Q30 bases distributed between 94.51% and 94.78%. The average GC content was 53.95%. By aligning reads to the reference genome, we obtained genome-wide alignment results for each sample, with alignment rates ranging from 97.5% to 97.8%. Differential analysis identified 228 differentially expressed mRNAs (DE-mRNAs), comprising 141 upregulated and 87 downregulated genes (Fig. 1A). These DE-mRNAs were predominantly distributed in the extracellular domain and significantly enriched in terms such as proteolysis, inflammatory and immune responses, chemokine-mediated signaling, antifungal humoral defense, and leukocyte proliferation regulation (Fig. 1A). KEGG functional enrichment analysis revealed a functional network centered on immune regulation, cell-cell communication, and metabolic reprogramming. Key pathways included cytokine-cytokine receptor interactions, IL-17 signaling, stem cell pluripotency regulation, and arrhythmogenic right ventricular cardiomyopathy (Fig. 1A). Differentially expressed long non-coding RNAs (DE-lncRNAs) analysis (Fig. 1B) identified 700 DE-lncRNAs, comprising 327 upregulated and 373 downregulated transcripts. These DE-lncRNAs were primarily localized to the extracellular region, extracellular space, plasma membrane, collagen matrix, and various granules, showing significant enrichment in terms such as proteolysis, calcium ion transport across membranes, inflammation and immune response, chemokine signaling, antifungal humoral defense, positive regulation of leukocyte proliferation, yeast response, and cell-cell communication KEGG functional enrichment analysis revealed these DE-lncRNAs were significantly enriched in pathways related to immune and inflammatory regulation, cell recognition and communication, energy metabolism, and arrhythmogenic right ventricular cardiomyopathy. Analysis of differentially expressed circRNAs (DE-circRNAs) (Fig. 1C) identified 1,188 DE-circRNAs, comprising 509 upregulated and 679 downregulated molecules. These DE-circRNAs were significantly enriched in terms such as transcription and gene expression regulation, organelle biology and stress response, immunity and antigen processing, metabolism and enzyme activity, and cell structure and repair. KEGG functional enrichment analysis revealed that these DE-circRNAs were significantly enriched in functions related to autoimmune and transplant immunity, infection and host defense, base excision repair, DNA replication, hematopoietic cell lineage differentiation, and metabolic regulation (Fig. 1C).

### GSEA of lncRNAs and PPI of mRNAs, lncRNAs, and circRNAs

**Fig. 2:**
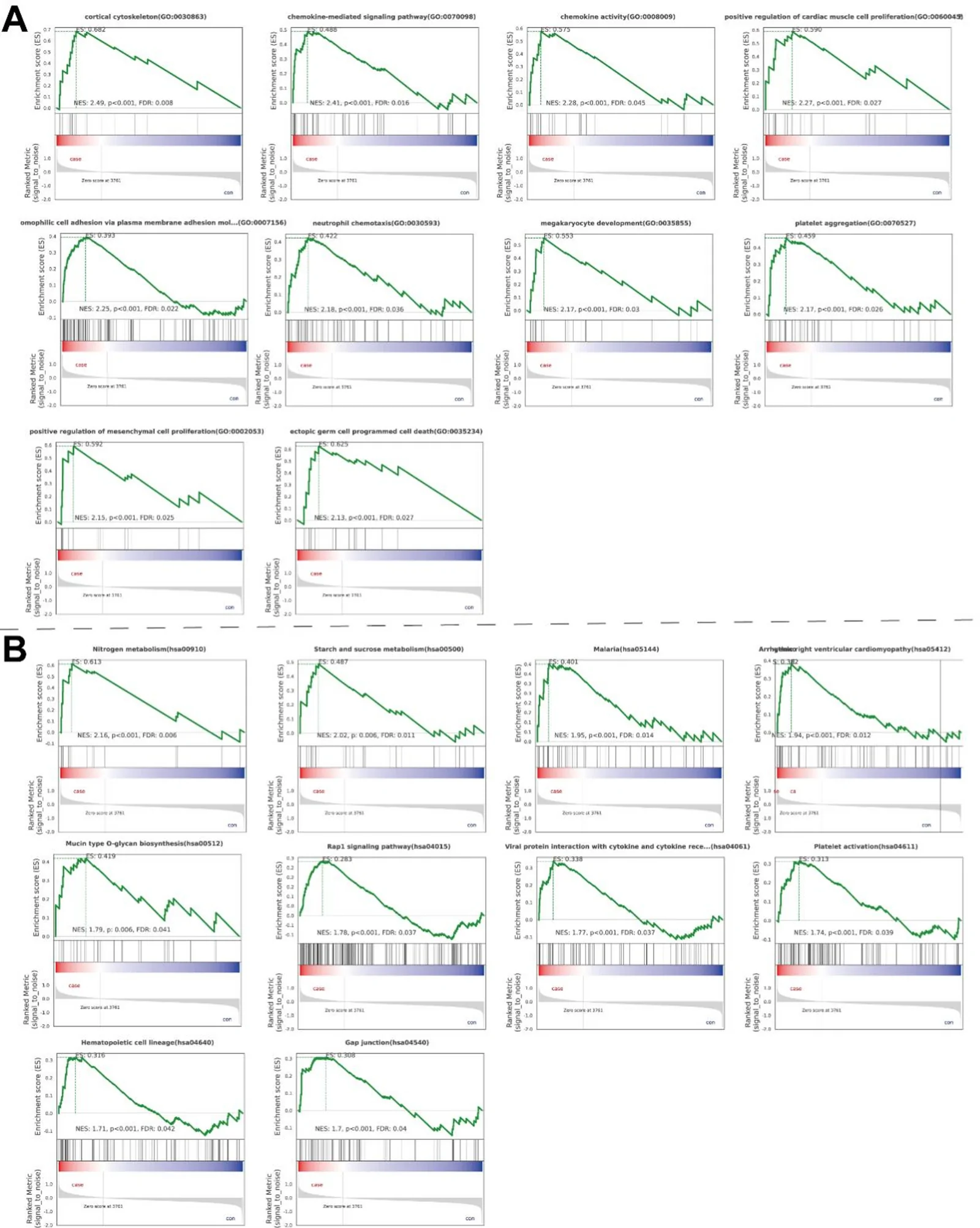
Top 10 GSEA analysis results for differentially expressed mRNAs in GO and KEGG. **A**: Top 10 GO Functional Enrichment in GSEA Analysis of Differentially Expressed mRNAs. **B**: Top 10 KEGG Functional Enrichment in GSEA Analysis of Differentially Expressed mRNAs. *n* = 5, Screening criteria: FDR q-value < 0.05, |NES| > 1.5

**Fig. 3:**
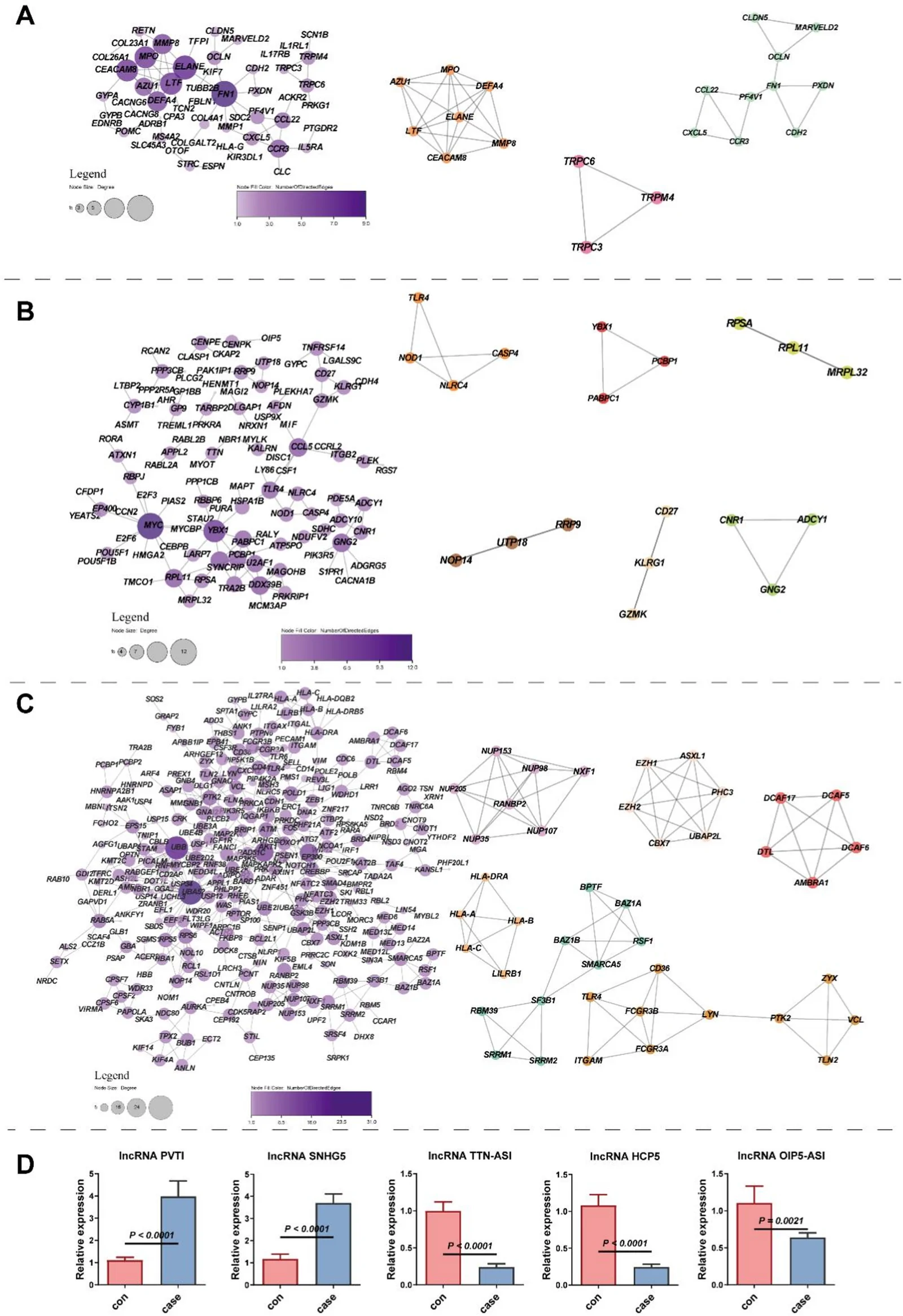
Protein-protein interaction network analysis of differentially expressed mRNAs, lncRNAs, and circRNAs, and validation of candidate key lncRNA expression. **A**-**C**: Protein-protein interaction network analysis of differentially expressed mRNAs (**A**), lncRNAs (**B**), and circRNAs (**C**), *n* = 5. **D**: Validation of key lncRNA expression by RT-qPCR, *n* = 3

GSEA analysis revealed that DE-mRNAs were significantly enriched in the cortical cytoskeleton (GO:0030863, NES: 2.49), chemokine-mediated signaling pathway (GO:0070098, NES:2.41), chemokine activity (GO:0008009, NES:2.28), and positive regulation of cardiac muscle cell proliferation (GO:0060045, NES:2.27) (Fig. 2A). Additionally, these DE-mRNAs were significantly enriched in Nitrogen metabolism (hsa00910, NES:2.16), Starch and sucrose metabolism (hsa00500, NES:2.02), Malaria (hsa05144, NES:1.95), and Arrhythmogenic right ventricular cardiomyopathy (hsa05412, NES:1.94) KEGG pathways (Fig. 2B). Additionally, a protein-protein interaction (PPI) network comprising 55 nodes and 66 interaction pairs was constructed based on DE-mRNA (Fig. 3A). Further analysis yielded three subnetwork modules with scores of 6.667, 3.000, and 2.889, respectively (Fig. 3A). Based on DE-lncRNA, a PPI network comprising 107 nodes and 114 interaction pairs was constructed (Fig. 3B). Further analysis yielded six subnetwork modules, with the first subnetwork scoring 3.333 and the remaining subnetworks scoring 3.000 (Fig. 3B). Based on DE-circRNA, a PPI network comprising 311 nodes and 533 interaction pairs was constructed (Fig. 3C). Further analysis yielded six subnetwork modules with scores of 6.333, 6.000, 5.000, 4.500, 4.250, and 4.222, respectively (Fig. 3C). Candidate key lncRNAs were identified by integrating DE-lncRNA differential expression and previous studies: lncRNA PVT1 (MYC), lncRNA SNHG5 (SYNCRIP), lncRNA TTN-AS1 (TTN), lncRNA HCP5 (MICB), and lncRNA OIP5-AS1 (OIP5). Expression validation revealed that PVT1 and SNHG5 were highly expressed in the myocardial infarction-induced heart failure group (case), while TTN-AS1, HCP5, and OIP5-AS1 were downregulated in the case group (Fig. 3D). These findings align with the transcriptome analysis results, corroborating the reliability of the transcriptomic assessment. 

### Construction of circRNA-mRNA and lncRNA-mRNA co-expression networks and ceRNA networks

**Fig. 4:**
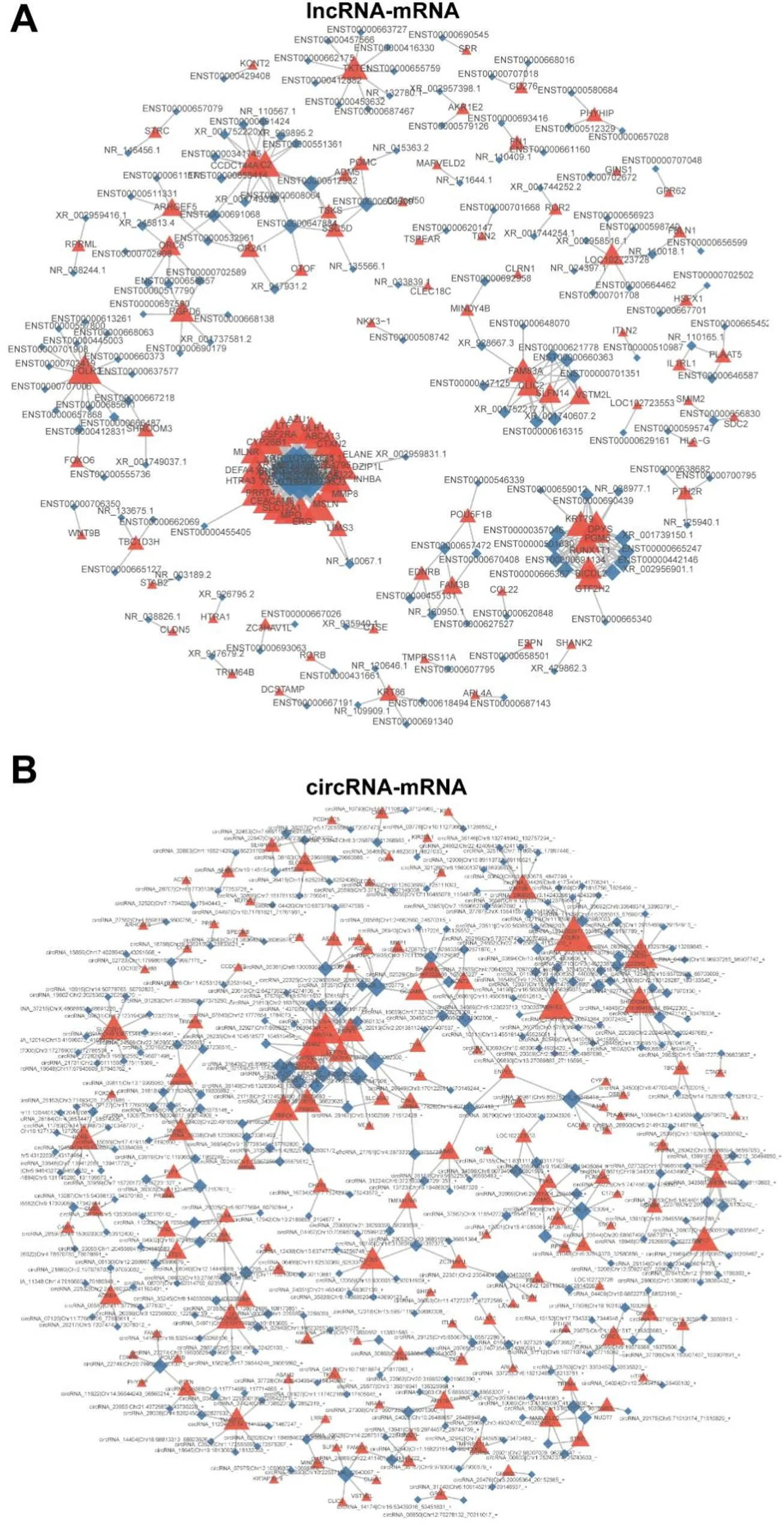
Interaction network analysis of lncRNA, circRNA, and mRNA. **A**: Interaction network of lncRNA, circRNA, and mRNA. **B**: Interaction network of circRNA and mRNA. Blue diamonds represent lncRNA or circRNA; red triangles represent mRNA

**Fig. 5:**
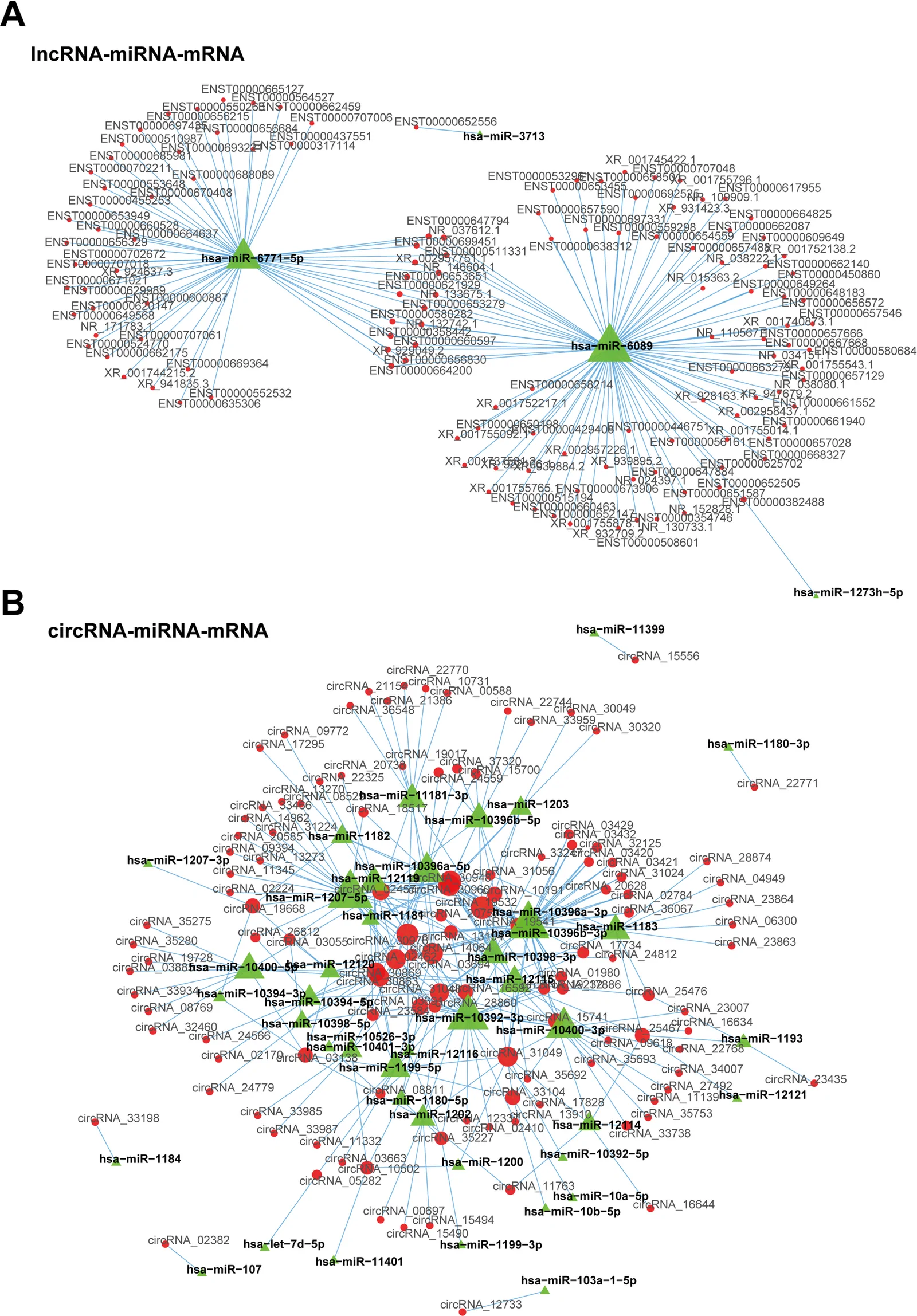
ceRNA network analysis. **A**: Construction of the lncRNA ceRNA network. **B**: Construction of the circRNA ceRNA network. Red circles represent lncRNAs or circRNAs, while green triangles represent miRNAs

This study comprehensively analyzed lncRNA-mRNA and circRNA-mRNA co-expression networks, as well as ceRNA networks constructed from lncRNA-miRNA-mRNA and circRNA-miRNA-mRNA interactions. Co-expression networks were constructed for the top 500 DE-lncRNA-mRNA pairs, as shown in Fig. 4A, comprising 365 downregulated pairs and 135 upregulated pairs. The co-expression network for the top 500 DE-circRNA-mRNA pairs is depicted in Fig. 4B, comprising 209 downregulated pairs and 291 upregulated pairs. The ceRNA network for the top 300 lncRNA-miRNA-mRNA pairs is shown in Fig. 5A, primarily involving four miRNAs: hsa-miR-3713, hsa-miR-6089, hsa-miR-6771-5p, and hsa-miR-1273 h-5p. Among these, hsa-miR-6089 formed 92 pairs and hsa-miR-6771-5p formed 56 pairs. The ceRNA network was constructed for the top 300 circRNA-miRNA-mRNA interaction pairs (Fig. 5B). The highly connected miRNAs in this ceRNA network were hsa-miR-1207-5p, hsa-miR-10392-3p, hsa-miR-10396a-3p, and hsa-miR-10396b-3p, comprising 27, 23, 23, and 21 relationship pairs, respectively.

### Analysis of lncRNA-transcription factor interactions

**Fig. 6:**
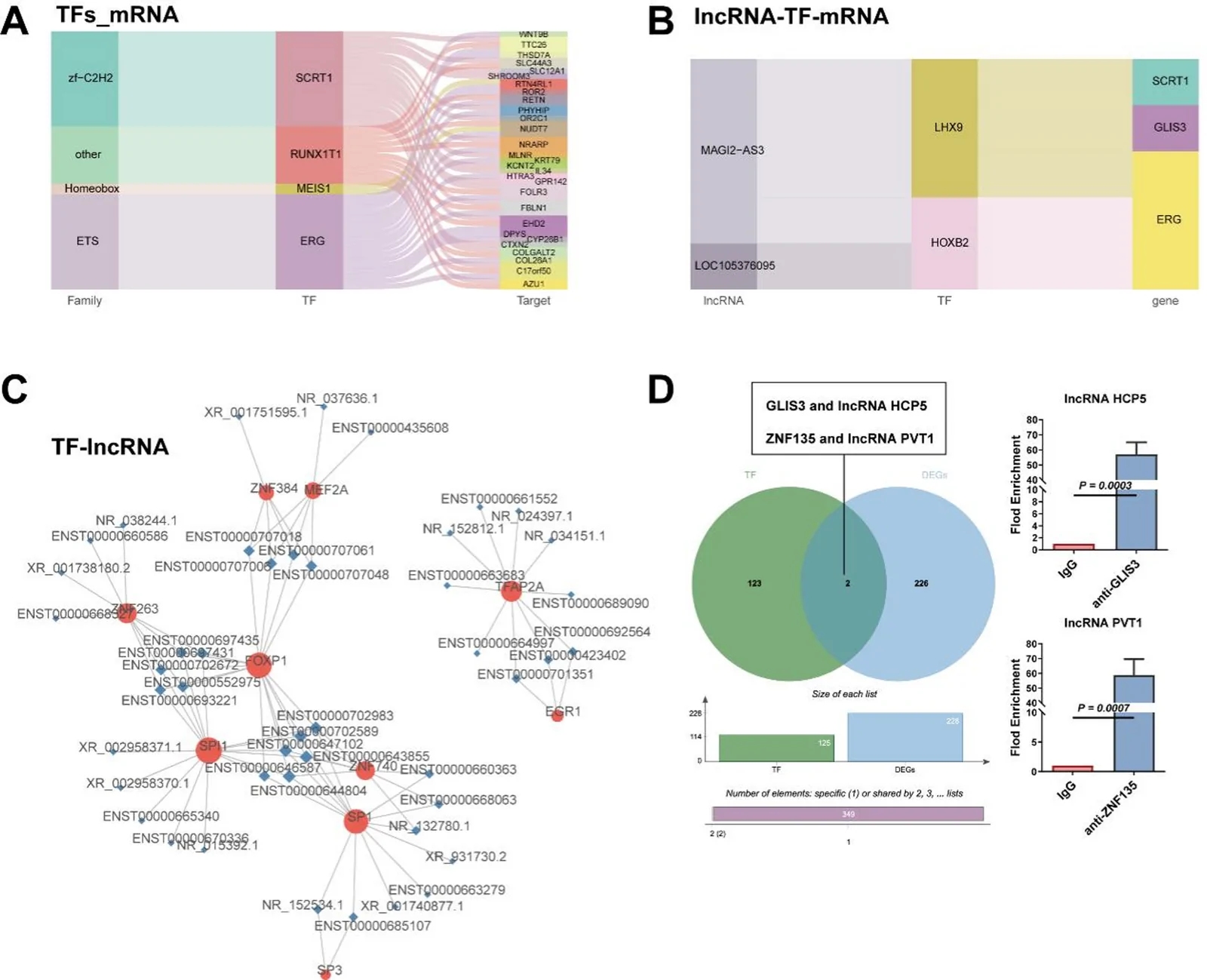
Transcriptional factor-lncRNA interaction analysis. **A**: Transcriptional factor-mRNA interaction analysis. **B**: lncRNA-transcriptional factor-mRNA interaction analysis. **C**: Transcriptional factor-lncRNA interaction network. **D**: Transcriptional factor-lncRNA intersection analysis and interaction validation via RIP-PCR

Given that lncRNAs can regulate transcription factor activity through multiple mechanisms (including chromatin remodeling, transcriptional regulation, and protein stability modulation), this study further constructed a lncRNA-transcription factor interaction network. Through transcription factor family enrichment analysis, multiple TF families were differentially activated in the heart failure group, and their target gene networks were identified. Notably, the zf-C2H2 zinc finger protein family exhibited significant target gene regulation activity with transcription factor SCRT1, the Homeobox family with transcription factor MEIS1, and the ETS family with transcription factor ERG (Fig. 6A). Based on the ceRNA mechanism, a three-tiered lncRNA-TF-mRNA regulatory network was constructed. The network core comprises two candidate key lncRNA nodes (MAG12-AS3 and LOC105376095) indirectly regulating two core transcription factors (LHX9, HOXB2) and their downstream target genes (SCRT1, GLIS3, ERG) through competitive miRNA binding (Fig. 6B). The TF-lncRNA transcriptional regulatory network exhibits a distinct hub-and-spoke architecture, identifying multiple core TF nodes and their regulated lncRNA clusters (Fig. 6C). Furthermore, Venn diagram analysis revealed only two overlaps among 125 differentially expressed TFs and 228 DE-lncRNAs: GLIS3-HCP5 and ZNF135-PVT1 (Fig. 6D). RIP-PCR experiments confirmed specific binding between GLIS3 and lncRNA HCP5 (enrichment factor ~ 57-fold, *P* = 0.0003) (Fig. 6D). Furthermore, the interaction between ZNF135 and lncRNA PVT1 was similarly validated (enrichment factor ~ 59-fold, *P* = 0.0007) (Fig. 6D).

## Discussion

This study analyzed the molecular mechanisms underlying the development of HF-MI through whole-transcriptome sequencing. It reveals the process from clinical phenotypes and transcriptomic features to multi-level regulatory networks, providing new insights into the pathophysiological mechanisms of post-MI heart failure.

This study first characterized the clinical features of HF patients after MI. The Case group exhibited significantly elevated NT-proBNP levels and markedly reduced LVEF%, consistent with the characteristics of heart failure patients [30]. Subsequently, whole-transcriptome analysis revealed differential expression patterns of mRNA, lncRNA, and circRNA in post-MI HF patients, each exhibiting distinct expression profiles and functional characteristics. DE-mRNA enrichment primarily targeted immune-inflammatory responses, chemokine signaling, and myocardial cell proliferation regulation, consistent with the pathological processes of early inflammatory infiltration and late myocardial remodeling following myocardial infarction [31]. Notably, DE-mRNA significantly enriched the arrhythmogenic right ventricular cardiomyopathy pathway, suggesting potential myocardial electrophysiological instability in post-MI HF patients [32], offering early warning for clinical arrhythmia prevention and treatment. The number of DE-lncRNAs and DE-circRNAs far exceeded that of DE-mRNAs, with a higher proportion of downregulation in the heart failure group compared to the non-heart failure group (lncRNA: 53.3%, circRNA: 57.2%), suggesting that ncRNAs may primarily exert negative regulatory effects during heart failure progression. Functional enrichment analysis revealed that lncRNAs are primarily involved in immune regulation and extracellular matrix remodeling [33, 34]. The notable enrichment of circRNAs in autoimmune and transplant immunity pathways suggests the potential existence of autoimmune responses against myocardial antigens post-MI, consistent with reports on autoimmune cardiomyopathy following myocardial infarction [35, 36].

Additionally, the ceRNA network plays a crucial role in ncRNA regulation of HF [12, 37]. This study constructed a multi-level regulatory system from the ceRNA network to the TF-lncRNA interaction network, revealing the systems biology characteristics of heart failure development. The ceRNA network analysis identified multiple key miRNA nodes, such as lncRNA and hsa-miR-6089, hsa-miR-1207-5p, circRNA and hsa-miR-1207-5p, hsa-miR-10392-3p. hsa-miR-1207-5p serves as a shared key miRNA in the ceRNA network formed by lncRNA and circRNA. Previous studies revealed that lncRNA participates in inflammation-induced injury regulation by modulating hsa-miR-1207-5p [38]. Additionally, hsa-miR-6089 was similarly identified as a differentially expressed miRNA in patients with coronary heart disease and hyperglycemia [39]. However, beyond these findings, these miRNAs have been sparsely reported in prior cardiovascular studies and may represent specific regulatory factors in post-MI heart failure, warranting further investigation. Importantly, this study constructed a multi-level interaction network between lncRNAs and transcription factors, experimentally validating two candidate key regulatory pairs: GLIS3-HCP5 and ZNF135-PVT1. Gli-similar-3 (GLIS3) is a multifunctional transcription factor whose core functions include regulating glucose metabolism by influencing β-cells [40], participating in inflammation-fibrosis activation [41], and immune modulation [42]. Importantly, GLIS3 is highly enriched in cardiomyocytes [43] and serves as a core regulatory transcription factor in smooth muscle cell phenotypic conversion during cardiovascular diseases [44]. HLA Complex P5 (HCP5), an immune-associated long non-coding RNA located within the MHC region, plays crucial roles in immune regulation, viral infection, tumor immunity, and cardiovascular diseases [45]. In cardiovascular disease-related studies, HCP5 sponge-mediated miRNA adsorption exerts cardioprotective effects [46]. This study reveals that GLIS3 binds to lncRNA HCP5, suggesting potential molecular-level crosstalk between metabolic disorders and immune inflammation in heart failure. HCP5 is downregulated in the heart failure group, while GLIS3, as a transcription factor, may potentially influence its stability or localization by binding to HCP5. Additionally, Zinc Finger Protein 135 (ZNF135), belonging to the zinc finger protein family, functions as a housekeeper tumor suppressor gene [47]. However, its cardiac role remains unclear. Plasmacytoma Variant Translocation 1 (PVT1) promotes tumor growth by stabilizing MYC protein and preventing its phosphorylation, acting as a key regulator in MYC-dependent tumors [48]. In cardiovascular disease research, PVT1 was found to be significantly upregulated in MI mice, participating in myocardial injury via sponge adsorption of miRNAs [49–51]. Similarly, this study observed high PVT1 expression in the heart failure group, coupled with binding to the transcription factor ZNF135. Notably, MYC functions as both a pro-proliferative and pro-apoptotic factor, and its dual role in cardiomyocytes may present a double-edged sword for heart failure therapy. The roles and mechanisms of action of the two-candidate key regulatory pairs, GLIS3-HCP5 and ZNF135-PVT1, in MI-induced HF warrant further investigation.

Although this study performed a full transcriptome analysis of HF-MI, it has several limitations. First, the small sample size (*n* = 5/group) allowed detection of significant differences but may have missed genes with weaker effects. The small sample size introduces several limitations to the analysis of clinical indicators, including false-negative results due to insufficient statistical power resulting from the small sample size, which necessitates validation in a larger sample. Second, the RIP experiment validated only two lncRNA-TF interaction pairs, requiring functional experiments to confirm additional predicted relationships. Moreover, Blood results cannot be directly equated with myocardial tissue expression. Future research will further verify cardiac specificity through paired samples (blood + myocardial biopsy/autopsy tissue). Furthermore, the cross-sectional design precludes determining whether these molecular alterations are causes or consequences of heart failure. Validation through further multicenter studies is essential to facilitate clinical translation of these findings.

## Conclusion

This study systematically mapped the molecular regulatory landscape of heart failure following myocardial infarction through whole-transcriptome sequencing and candidate bioinformatics analysis, revealing a multi-level regulatory network centered on lncRNA-transcription factor interactions. Experimental validation of two candidate key regulatory pairs—GLIS3-HCP5 and ZNF135-PVT1—provides new insights into the epigenetic-transcriptional co-regulation mechanisms underlying heart failure. These findings not only enrich the pathophysiological theory of heart failure but also lay the foundation for developing novel diagnostic candidate biomarkers and therapeutic targets.

## Data Availability

Data will be made available by the corresponding author on request. The datasets generated and/or analysed during the current study are available in the NCBI GEO repository, accession number: GSE324415.

## Abbreviations

AMI: Acute myocardial infarction
HF: Heart failure
ncRNAs: Non-coding RNAs
lncRNAs: Long non-coding RNAs
circRNAs: Circular RNAs
miRNAs: microRNAs
ceRNAs: Competitive endogenous RNAs
NHF-MI: AMI without heart failure
HF-MI: AMI complicated by heart failure
DE: Differentially expressed
LVEF: Left ventricular ejection fraction
NT-proBNP: N-terminal pro-B-type natriuretic peptide
GO: Gene Ontology
KEGG: Kyoto Encyclopedia of Genes and Genomes
GLIS3: Gli-similar-3
HCP5: HLA Complex P5
ZNF135: Zinc Finger Protein 135
PVT1: Plasmacytoma Variant Translocation 1
